# Marion Elizabeth Blackman

**Published:** 1915-10

**Authors:** 


### Additional Obituary.

**Marion Elizabeth Blackman**, born at Worcester, Mass, Feb. 7, 1878, the daughter of a physician, attended schools in Brooklyn and graduated at the Girls' High School at Brooklyn. Graduated in medicine at the University of Michigan in 1909 and served interneship at the New England Hospital for Women and Children at Boston. She practiced for a time in Wyoming County and then held, for several years, a position on the staff of the Rochester State Hospital, until the beginning of an illness about three years before her death. Recently she had been at Castile, N. Y. She died in Buffalo, September 3, 1915.

